# Investigation of lncRNA in *Bos taurus* Mammary Tissue during Dry and Lactation Periods

**DOI:** 10.3390/genes14091789

**Published:** 2023-09-12

**Authors:** Alexis Marceau, Junjian Wang, Victoria Iqbal, Jicai Jiang, George E. Liu, Li Ma

**Affiliations:** 1 Department of Animal and Avian Sciences, University of Maryland, College Park, MD 20742, USA; amarceau@umd.edu (A.M.); torip96@umd.edu (V.I.); 2 Department of Animal Science, North Carlonina State University, Raleigh, NC 27695, USA; jwang259@ncsu.edu (J.W.); jicai_jiang@ncsu.edu (J.J.); 3 Animal Genomics and Improvemennt Laboratory, BARC, USDA-ARS, Beltsville, MD 20705, USA; george.liu@usda.gov

**Keywords:** *Bos taurus*, lncRNA, mammary

## Abstract

This study aims to collect RNA-Seq data from *Bos taurus* samples representing dry and lactating mammary tissue, identify lncRNA transcripts, and analyze findings for their features and functional annotation. This allows for connections to be drawn between lncRNA and the lactation process. RNA-Seq data from 103 samples of *Bos taurus* mammary tissue were gathered from publicly available databases (60 dry, 43 lactating). The samples were filtered to reveal 214 dry mammary lncRNA transcripts and 517 lactating mammary lncRNA transcripts. The lncRNAs met common lncRNA characteristics such as shorter length, fewer exons, lower expression levels, and less sequence conservation when compared to the genome. Interestingly, several lncRNAs showed sequence similarity to genes associated with strong hair keratin intermediate filaments. Human breast cancer research has associated strong hair keratin filaments with mammary tissue cellular resilience. The lncRNAs were also associated with several genes/proteins that linked to pregnancy using expression correlation and gene ontology. Such findings indicate that there are crucial relationships between the lncRNAs found in mammary tissue and the development of the tissue, to meet both the animal’s needs and our own production needs; these relationships should be further investigated to ensure that we continue to breed the most resilient, efficient dairy cattle.

## 1. Introduction

Although many research endeavors have placed emphasis on direct gene products, sequencing technology advancements have led to a shift in focus. Researchers have now taken on investigating the regions of the genome that are not made into proteins: non-coding RNA. Non-coding RNA has been established as a gene expression regulator in many roles, including (but not limited to) X chromosome inactivation, allelic imprinting, pluripotency control, and cancer [1]. An important group within the non-coding RNA family is the long noncoding RNA (lncRNA): non-coding transcripts that are longer than 200 nucleotides. Research has shown that these transcripts are far-reaching and common, with more than 270,000 lncRNAs identified in humans, approximately 21,000 lncRNAs found in mice, and over 7235 lncRNAs present in cattle [2,3,4]. Not only are lncRNAs found in many organisms, but they are also found in variable numbers in subspecies and breeds. In previous studies within two Chinese cattle breeds (and across six tissues), over 4000 lncRNAs were identified; an additional study utilizing 18 different bovine tissue uncovered almost 10,000 transcripts; tissue-specific studies found over 23,000 lncRNAs in bovine testes over maturation and over 1500 transcripts present in bovine oocytes [5,6,7,8]. In addition to lncRNA being found in many tissues, they have been linked to biological processes: for example, nearly 8000 lncRNAs were associated with cattle metabolic efficiency [9]. Given that lncRNAs are so wide-reaching and are under-researched, investigating the role of lncRNAs in mammary tissue may reveal more about the biological processes behind lactation, a biologically significant and economically crucial process in a dairy cow’s life.

Between birth and puberty, the mammary gland (which includes the nipple, areola, stroma, and parenchyma) is and remains primarily underdeveloped. The surging levels of hormones lead the mammary tissue to increase in size during puberty, but this growth does not proceed past the filling of the mammary fat pad at which time, and the duct network has begun to branch but is not fully developed [10]. Gestation leads to the final developmental push in the mammary gland where the parenchyma increases in size and its duct system finishes branching. The duct terminals become surrounded by connective tissue and develop into secretory alveoli. At the end of pregnancy, the ducts fill with milk and the animal is ready to nurse its offspring, fulfilling the mammary gland’s primary function: milk secretion. If an animal does not become pregnant and does not receive the pregnancy-based surge of estrogen and progesterone, the mammary gland development remains arrested [11].

As expected with such large physical changes, the development of the mammary gland is associated with changes in genetic expression. An example of this came from an investigation into puberty’s genetic effect in cattle; researchers found 82 transcription factors in liver tissue that influence widespread tissues, demonstrating that the developmental process is reliant on gene expression changes [12]. When examining heat stress in cattle (relating to milk production), over 3000 genes were identified in the mammary gland alone, relating to anabolism, milk component synthesis, cell death, cytoskeleton degradation, and immune response, giving further support to the importance gene expression plays in mammary function [13]. Given the evidence of the genetic components of puberty and mammary gland tissues, and the vast number of morphological changes in mammary gland tissues, clearly there are many genetic elements at play in the development of mammary tissue.

Given the ever-increasing demand for dairy products, researchers and farmers alike have spent decades selectively breeding cows that produce the most and best milk possible. And, although there are well-researched genes involved in the amount and composition of milk, finding which genes are present and activated by lactation is a different beast. Research has uncovered 881 differentially expressed genes in the mammary gland tissue of lactating cows versus dry cows. The genes present in lactating cows are related to metabolic processes, protein synthesis, growth processes, immune-related processes, and more! These findings led researchers to conclude that dry cows had decreased capacity for protein synthesis, energy generation, and cell growth. However, dry cows may have a stronger immune response when compared to their lactating counterparts. Given the considerable number of coding genes identified as present between dry versus lactating cows, there is likely to be a universe of non-coding elements that are also at play [14].

Here, we report a genome-wide investigation into lncRNA isolation and identification within mammary tissue in dry versus lactating *Bos taurus* specimens. Using well-tested tools, samples were sequenced, aligned, and filtered based on lncRNA requirements. Identified lncRNA were then analyzed based on differential expression, sequence conservation, orthologous genes, GWAS enrichment, and expression correlation-based gene ontology. By uncovering more information about the lncRNA present in these tissues and their expression profiles, we both increased the knowledge about lncRNA and their connection to production traits and animal development.

## 2. Materials and Methods

### 2.1. Data Collection

The SRA database was parsed for rumen tissue samples from *Bos taurus* animals, searching for both dry and lactating samples. In total, 60 samples were found for dry tissue and 43 samples were found for lactating tissue, and SRR IDs were recorded.

Data were downloaded locally using the SRA Toolkit, using the fastq-dump tool. The split-3 option was used to ensure all reads in the raw data were collected [15]. SRA Toolkit: https://github.com/ncbi/sra-tools (accessed on 1 October 2022).

### 2.2. Sequence Assembly and Mapping

RNA-Seq data were mapped to the ARS-UCD1.2 reference genome using the spliced read aligner Hisat 2.2.1, after the ARS-UCD1.2 reference genome fasta file was used to build the necessary index file [16,17]. Mapping was performed twice; the first approach was used to generate a junction file representing junctions between transcripts, and the second iteration used those generated junctions to increase fidelity of the mapping results. Mapped transcripts were then stitched into an assembled report for each sample using Stringtie v2.2.1. This was performed twice: once using the ARS-UCD1.2 genome and a second time with the UMD3.1.1 reference genome, for additional accuracy [18,19]. Samples were then ready for further analysis. Hisat2: http://daehwankimlab.github.io/hisat2/ (accessed on 1 October 2022). Stringtie: https://ccb.jhu.edu/software/stringtie/ (accessed on 1 December 2022). ARS-UCD1.2 genome: https://www.ncbi.nlm.nih.gov/datasets/genome/GCF_002263795.1/ (accessed on 1 October 2022). UMD3.1.1 genome: https://www.ncbi.nlm.nih.gov/datasets/genome/GCF_000003055.6/ (accessed on 1 October 2022).

### 2.3. lncRNA Identification

Long non-coding RNA (lncRNA) identification started with using CuffCompare 2.2.1 software to annotate transcripts based on known genetic features [20]. Summary files representing samples aligned to the ARS-UCD1.2 reference genome were compared to the ARS-UCD1.2 reference genome, and summary files representing samples aligned to the UMD3.1.1 reference genome were compared to the UMD3.1.1 reference genome. Transcripts returning the intergenic “-u” flag were isolated, generating a list of intergenic transcripts for all summary files. For each sample, the list of intergenic transcripts from the ARS-UCD1.2 and UMD3.1.1 were combined and only unique transcripts retained, generating a summary list of transcripts deemed intergenic based on 2 reference genomes. Intergenic lists for all dry samples were merged to generate a single intergenic list for dry tissue; this was repeated for lactating samples. Those transcripts that were shorter than 200 basepairs in length were removed at this point. Intergenic transcripts were inputted into the Coding Potential Coding (CPC), where they were scored based on open reading frame and homology analysis, receiving a score between −1 and 1 [21]. Those transcripts that scored below a 0 were deemed non-coding, and the reverse compliment of all intergenic transcripts was also analyzed with the CPC, allowing for generation of a list of intergenic transcripts deemed non-coding in both directions. Non-coding, intergenic transcripts were converted into protein sequences for each transcript and sequences were compared to the Pfam database to remove those that demonstrated known protein domains [22]. Remaining transcripts were returned to their genomic location coordinates, and the sequences associated with those coordinates were compared to known gene sequences to remove those transcripts that appeared to be known genes based on sequence homology using BLAST [23]. After all filtering steps, lists of transcripts remained for dry and lactating tissue that contained only transcripts that were: (a) intergenic, therefore not overlapping known genes; (b) longer than 200 basepairs in length; (c) did not show coding potential; (d) did not contain a known protein domain; and (e) did not have notable sequence homology with known genes. These remaining transcripts match lncRNA criteria. CuffCompare: http://cole-trapnell-lab.github.io/cufflinks/cuffcompare/ (accessed on 1 December 2022). Coding Potential Calculator: http://cpc2.gao-lab.org/ (accessed on 1 December 2022). Pfam: https://www.ebi.ac.uk/interpro/ (accessed on 1 January 2023). BLAST: https://blast.ncbi.nlm.nih.gov/Blast.cgi?PROGRAM=blastn&PAGE_TYPE=BlastSearch&LINK_LOC=blasthome (accessed on 1 January 2023).

### 2.4. Comparison to Coding Transcripts

Length was calculated for all transcripts and all lncRNA in both conditions. Average length was also calculated for each profile. This allowed for comparison of transcript length profiles. All transcript length data and lncRNA transcript length data were pooled, and these values were graphed in a histogram using R4.2.2, with the addition of an average length representative line [24]. A truncated graph was also created to add clarity.

Expression data were generated for all transcripts in all samples using the—A option of Stringtie v2.2.1 [18]. This is carried out via read clustering, leading to the creation of a splice graph for each read cluster that is associated with a transcript, creating a flow network for each transcript, allowing for estimation of expression level using a maximum flow algorithm, as measured in fragments per kilobase of transcript per million mapped reads (FPKM). Similarly to length profiles, this allowed for generation of expression profiles for both conditions, representing all transcripts and lncRNA. The average expression level was calculated. Expression data for all transcripts in both conditions and all lncRNAs in both conditions were pooled and charted in a boxplot using R, and expression profiles for all profiles were also graphed in a boxplot using R.

Stringtie v2.2.1 also reports exons per transcript, allowing for calculation of exon number profiles for all transcripts in both conditions and lncRNAs in both conditions [18]. These values were reported. R: https://www.r-project.org/ (accessed on 1 March 2023).

### 2.5. Differential Expression Analysis

Expression profiles for common transcripts were isolated from lncRNA expression profiles for both conditions. R was used to perform a two-sample *t*-test or a Welch’s *t*-test, based on variance, where transcripts were deemed statistically significantly differentially expressed when the reported *p*-value was less than 0.05. Statistically significant results were highlighted in green for clarity.

### 2.6. Phastcon Analysis

Sequence conservation was investigated using a Phastcon-based analysis to assign a conservation score to regions of interest. Using transcript profiles for all transcripts, intergenic transcripts (as generated by CuffCompare), and lncRNA transcripts in both conditions, the UCSC LiftOver webtool was used to convert ARSUCD1.2 cattle genome coordinates to hg38 human genome coordinates [25]. The multiBigwigSummary tool was used in conjunction with the hg38 100-way conservation alignment table to generate a conservation score for each provided region [26,27]. The hg38 100-way table utilizes data from 100 species ranging from simple lamprey to human. Data for both conditions for all transcripts, intergenic transcripts, and lncRNAs were pooled and graphed in a boxplot using R. Conservation scores for each profile were graphed in a violin plot using R as well. lncRNAs from dry and lactating tissues were assigned a rank based on their relative conservation score and this rank was graphed against the conservation score using R. lncRNAs whose conservation score was above a 0.5 were retained for further analysis.

LiftOver: https://genome.ucsc.edu/cgi-bin/hgLiftOver (accessed on 1 January 2023). MultiBigWigSummary: https://deeptools.readthedocs.io/en/develop/content/tools/multiBigwigSummary.html (accessed on 1 March 2023).

### 2.7. Transcriptional Annotation Based on Sequence Homology

All common lncRNA transcripts and those lncRNAs whose conservation score was above 0.5 were retained for transcriptional annotation, using their hg38 genome locations. Genomic regions were seared in the UCSC genome browser and overlapping genes were noted. Once all candidate regions were searched, overlapping genes were investigated with UniProt to assign a function to each gene. Patterns and common functions were notated and assessed based on findings. UCSC Genome Browser: https://genome.ucsc.edu/ (accessed on 1 March 2023). UniProt: https://www.uniprot.org/ (accessed on 1 March 2023).

### 2.8. Gene Co-Expression Correlation and Ontology

Salmon was used to quantify the expression of transcripts in all transcript profiles and lncRNA profiles for both conditions, reporting transcript per million (TPM) data for all transcripts provided within all samples. Salmon’s additional function, quantmerge, was used to combine expression data to create summary files for all transcripts for both samples, with lncRNA being explicitly labeled [28,29].

To eliminate false co-expression relationships from being generated due to low expression, any transcript lacking expression in at least 5 samples was removed from both summary files. Once these filtered expression summary files were created, a correlation matrix was generated for both tissue conditions using the corr function in R. These matrices were then filtered to only reflect correlations with lncRNAs, excluding gene-to-gene based correlations. These values were retained for reporting. The matrices were then converted to *p*-values, also using R.

Using excel for its ease of column sorting, each lncRNA was sorted based on ascending *p*-value. IDs of those genes that correlated with lncRNA with a *p*-value less than 0.05 were retained and passed to the web-based gene ontology enrichment analysis tool, parsing for biological process, limiting results to *Bos taurus* [30]. The result showing the highest hierarchical rank was noted, retaining biological process, fold enrichment value, raw *p*-value, and false discovery rate data. After all retained lncRNAs in both tissue conditions were analyzed for gene ontology, the results were able to be analyzed for pattern and significance. Salmon: https://combine-lab.github.io/salmon/ (accessed on 1 April 2023).

### 2.9. Transcriptional Annotation Based on Correlation

Using the correlation matrices generated for gene co-expression analysis, the gene with the most significant *p*-value for each investigated lncRNA was further investigated. This was carried out by parsing the Ensembl gene database, noting the most significantly associated gene for each lncRNA. The function of each gene was assessed using the NCBI gene database. Most functions were defined by their role in humans as more information is available in human-based reports; however, the roles appear to be non-species specific and gene names were identical.

Once all gene functions were noted, patterns were assessed and reported.

### 2.10. Heritability Enrichment Analysis

To complete a heritability enrichment analysis, two sets of lncRNA were generated: those present in dry tissue and those present in lactating tissue. GWAS data obtained from 27,000 bulls and 3 million SNPs/INDELs were used to assess cattle production (milk), reproduction (daughter pregnancy rate), health (livability), and body conformation (stature) [31,32]. Imputation of the SNPs allowed for reliable predicted transmitting abilities (PTAs) for the represented traits. This was carried out using MPH for enrichment analysis [33]. MPH uses the following linear mixed model with two genetic components to fit the data:

$$y=Xb+g_{1}+g_{2}+e$$

$$g_{1}∼N\left(0,G_{1}\sigma_{g}^{2}1\right)$$

$$g_{2}∼N\left(0,G_{2}\sigma_{g}^{2}2\right)$$

$$e∼N(0,R\sigma_{e}^{2})$$

The variables are measured as follows:

*b*: a vector of fixed effects including intercept
*X*: a covariate matrix corresponding to *b*, $g_{!}$, and $g_{2}$ $g_{1}$: a vector of random effects representing the genetic contributions of the variants within lncRNAs
$g_{2}$: a vector of random effects representing the genetic contributions of the variants within the remaining genome
*e*: a vector of residual effects
$G_{1}$: a genomic relationship matrix (GRM) constructed by genotypes corresponding to lncRNAs
$G_{2}$: a genomic relationship matrix (GRM) constructed by genotypes corresponding to the remaining genome

*G* was computed using the following formula:

$$G=\frac{ZZ^{T}}{m}$$

where *Z* represents the standardized genotypes, *m* is the number of variants in the GRM, using $m_{1}$ and $m_{2}$ to represent the number of variants within lncRNAs and the remaining genome, respectively. *R* is a diagonal matrix used to model individual reliability of deregressed PTAs. The method of calculation used by MPH relies on a Monte Carlo REML algorithm that estimates variance components (VCs) and uses a Fisher information matrix to compute the variance–covariance matrix of VC estimates. $\frac{\sigma_{g}^{2}1}{m_{1}}$ equals per-SNP genetic variation in lncRNA, allowing for computation of per-SNP heritability enrichment using $\rho =\frac{\frac{\sigma_{g}^{2}1}{m_{1}}}{\frac{\sigma_{g}^{2}1+\sigma_{g}^{2}2}{m_{1}+m_{2}}}$. A delta method was used to calculate the standard error of ρ.

For the 731 lncRNAs (214 dry lncRNAs, 517 lactating lncRNAs), each transcript was analyzed with a 10 kb extension window on both sides, testing for their enrichment of per-SNP heritability for the 4 representative traits of interest. This extension window was used to encapsulate linked regions, based on SNP density within GWAS data and linkage disequilibrium levels in the population (46–48). Enrichment analysis was performed with nearly 3 million autosomal variants, with a threshold of a minimal MAF value of 0.005 and a HWE *p*-value of at least $1\times 10^{-8}$. A total of 4257 variants and 12,884 variants were identified within dry and lactating lncRNAs, respectively. SNP heritability was partitioned into two genomic relationship matrices (GRMs), one representing SNPs and INDELs within lncRNAs, and the other representing the remaining SNPs/INDELs. Enrichment was measured as the ratio of per-SNP heritability near lncRNAs as compared to the genome-wide heritability, where MPH reported enrichment value and standard error. *p*-value was computed using a *t*-test. MPH: https://jiang18.github.io/mph/index (accessed on 1 June 2023).

## 3. Results

### 3.1. lncRNA Identification

**Figure 1:**
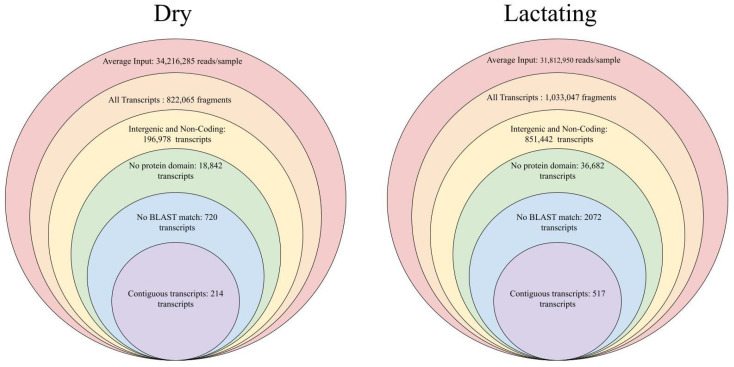
Filtering of transcripts in dry and lactating mammary tissue samples. After a consensus sequence was generated for each condition type, progressive filtering steps were taken to remove transcripts that do not meet lncRNA transcript criteria. Transcripts were aligned to the reference genome, transcripts were then kept if they were deemed intergenic based on the reference genome, noncoding as determined by the Coding Potential Calculator, lacking a definable protein domain, and lacking sequence homology. The filtering resulted in 214 contiguous transcripts in the dry tissue and 517 contiguous transcripts in the lactating tissue.

To generate a large data set, the SRA database was used to collect a total of 103 samples, with 60 samples representing dry mammary tissue and 43 samples representing lactating mammary tissue, all from *Bos taurus* [34]. Dry mammary tissue samples resulted in over 2 billion individual reads, with an average of 34,216,285 reads per sample. Lactating mammary tissue samples totaled 1.4 billion reads, averaging 31,812,950 reads per sample. Double-iterative mapping was used on all samples via Hisat2, generating an overall alignment rate of 89.61% for dry samples and 94.09% for lactating samples [16]. Using Stringtie to generate a merged, consensus sequence that was representative of each condition led to over 1 million fragments, representing 78,449 transcripts in the dry samples, and approximately 822,000 fragments, representing 100,239 transcripts in the lactating samples [18]. Given the increase in functionality of the mature mammary tissue, it is not surprising to see more transcripts represented in the lactating tissue. Once consensus sequences were generated, they were analyzed and filtered to remove fragments that did not meet lncRNA qualifications (meaning transcripts were longer than 200 basepairs in length and did not show evidence that they were made into a protein). First, consensus sequences were compared to both the ARS-UCD 1.2 *Bos taurus* reference genome and the UMD 3.1.1 *Bos taurus* reference genome to annotate known loci and transcripts, allowing their removal. This was followed by removal of transcript fragments that showed no coding potential as calculated by the coding potential calculator [35]. Noncoding transcripts were converted from sequence data to protein strings and fed through the protein family database to remove those with substantial protein domains within their sequence [22]. The final filtering step consisted of using BLAST to remove sequences that showed high sequence homology to known genes [23]. Once a final list of candidate fragments were generated, they were merged based on overlap to generate contiguous transcripts using the bedtools merge function [36]. The final list of lncRNAs for dry mammary tissue included 214 non-overlapping transcripts and 517 non-overlapping transcripts for lactating mammary tissue (Figure 1). Similarly to the pattern seen when observing all genome transcripts, the mature tissue has more functionality; therefore, it possesses more transcripts.

### 3.2. lncRNAs as Compared to Protein Coding Transcripts

**Figure 2:**
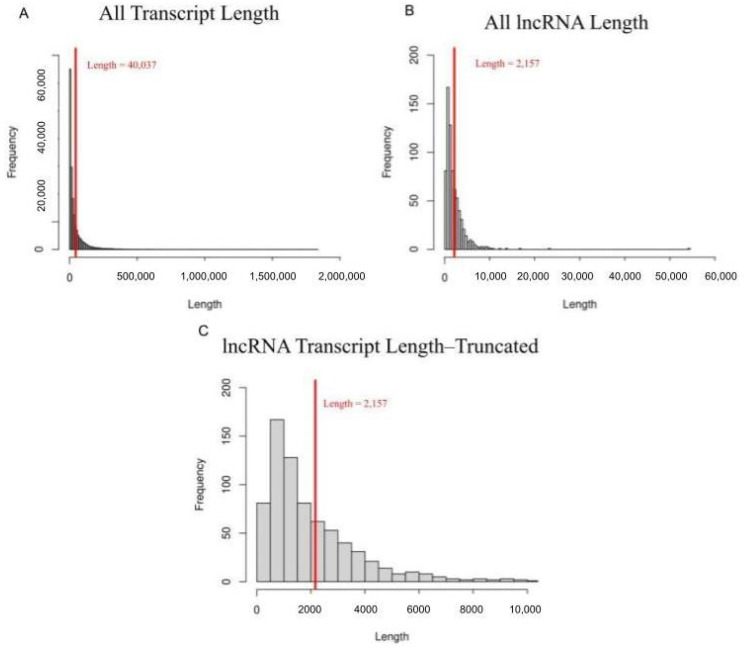
Length distribution of various transcripts. (**A**) Length of all transcripts in both dry and lactating samples. Transcripts ranged from 13 to 1,838,491 basepairs in length, the red line indicates the average transcript length of 40,037 basepairs. (**B**) Length of lncRNA transcripts in both dry and lactating samples, ranging from 202 to 16,503 basepairs. The average transcript length for candidate lncRNAs is 2157 bases and is indicated by the red line. (**C**) For added clarity, a truncated histogram representing lncRNA lengths, excluding the small number of those longer than 10,000 basepairs. Red line remains indicative of the average lncRNA length of 2157 basepairs.

When analyzing the entire genome, dry transcripts ranged from 53 to 1,525,771 bases, with the average length being 42,201 basepairs. Dry lncRNAs ranged from 210 to 16,503 bases, averaging 2139 bases. This fits with previous research demonstrating that even with the 200-basepairs minimum length, lncRNAs are characteristically shorter than their whole genome counterparts. These findings continued into lactating transcripts, which ranged from 13 basepairs in length to 1,838,491 basepairs, with the average length 37,873 reads in the whole genome compared to the 202 to 54,234 bases seen in lactating lncRNA, which averaged 2175 bases (Figure 2). Another key characteristic of lncRNA is the lower number of exons per transcript [37]. Dry whole genome transcripts averaged 7.912 exons per transcript and lactating whole genome transcripts averaged 6.5 exons per transcript. Dry lncRNAs showed 1.6 exons per transcript and lactating lncRNAs showed 2.2 exons per transcript. Both the length and exon number findings support the conclusion that the lncRNAs identified are in fact lncRNAs and not false positives.

**Figure 3:**
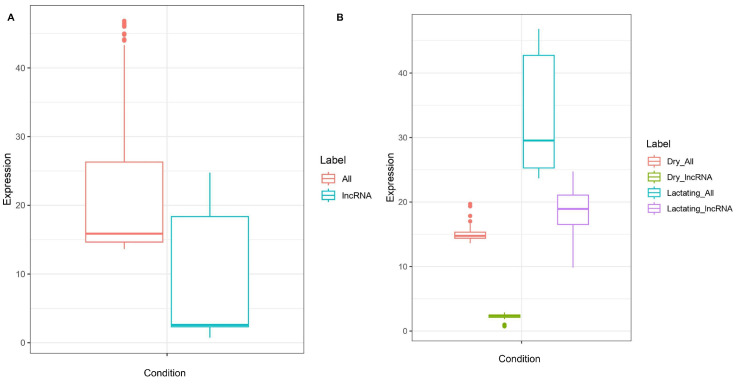
Expression of transcripts. (**A**) Using FPKM values, lncRNA are expressed at demonstrably lower levels than coding transcripts. All transcripts averaged 24.36 FPKM. lncRNA averaged 10.52 FPKM. (**B**) Expression levels additionally divided by condition type, measured via FPKM. Pattern of lower lncRNA expression as compared to all transcripts continues to hold true. Dry coding and lncRNA transcripts show lower expression than lactating transcripts, regardless of transcript type. Dry lncRNAs averaged 2.239 FPKM, all dry transcripts averaged 15.076 FPKM, lactating lncRNAs averaged 18.794 FPKM, and all lactating transcripts averaged 33.595 FPKM.

Another indicator in favor of candidate lncRNAs being genuine is the expression level. It is common to see lncRNAs show lower expression levels as compared to their whole genome and/or protein coding counterparts [38]. Overall, dry transcripts were expressed at an average level of 15.076 FPKM and lactating transcripts showed 33.595 FPKM expression levels on average. Dry lncRNA transcripts were expressed at 2.239 FPKM on average, lactating lncRNAs were expressed at 18.794 FPKM. Although not particularly proportional, the theme of lactating transcripts being expressed at a higher level is seen in both all transcripts and lncRNAs. The levels of expression are demonstrably higher in all transcripts as compared to lncRNAs only (Figure 3).

### 3.3. Differential Expression of lncRNA in Dry Versus Lactating Mammary Tissue

Of the 214 dry lncRNA transcripts and the 517 lactating lncRNA transcripts, there were 56 transcripts that covered similar regions of the genome. In total, 3 were removed due to expression not being seen in at least ½ of the samples in each condition. These lncRNA transcripts ranged from 229 basepairs to 16,503 basepairs, with dry lncRNAs averaging 2343 reads in length and lactating lncRNAs averaging 2454 basepairs. These lengths are close to the lengths seen in all lncRNAs (2139 bases in all dry lncRNAs, 2175 bases in lactating lncRNAs). The expression levels of common lncRNAs was 7.778 in dry lncRNAs and 6.747 in lactating lncRNAs. Of the 56 common lncRNAs, 44 transcripts were differentially expressed at a statistically significant level at *p* < 0.05, when compared with a student *t*-test. In total, 22 of the differentially expressed transcripts were upregulated in the dry tissue and 22 were upregulated in the lactating tissue. The final 9 common transcripts were not differentially expressed at a statistically significant level (Table 1).

### 3.4. Sequence Conservation of lncRNA

To continue the validation of candidate transcripts, as well as to begin to attempt to assign roles to the transcripts of interest, sequence conservation was investigated. This was carried out by using the UCSC LiftOver tool to lift over all transcripts, solely intergenic transcripts, and lncRNA transcripts to their matching coordinates on the human genome. A Phastcon 100-way file was used to determine the level of sequence conservation across lncRNA-associated regions of interest [39].

**Figure 4:**
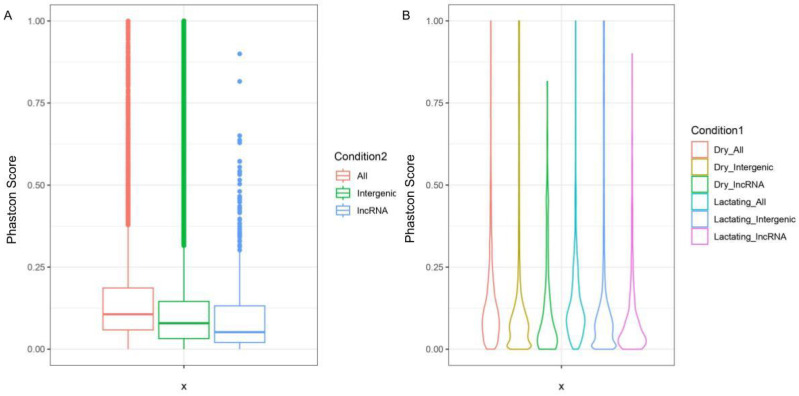
PhastCons scores of various transcript groups. (**A**) Boxplot of PhastCons scores for all transcripts, intergenic transcripts, and lncRNA transcripts. (**B**) Violin plot of all transcript profiles: all dry transcripts, dry intergenic transcripts, dry lncRNA transcripts, all lactating transcripts, lactating intergenic transcripts, and lactating lncRNA transcripts.

**Figure 5:**
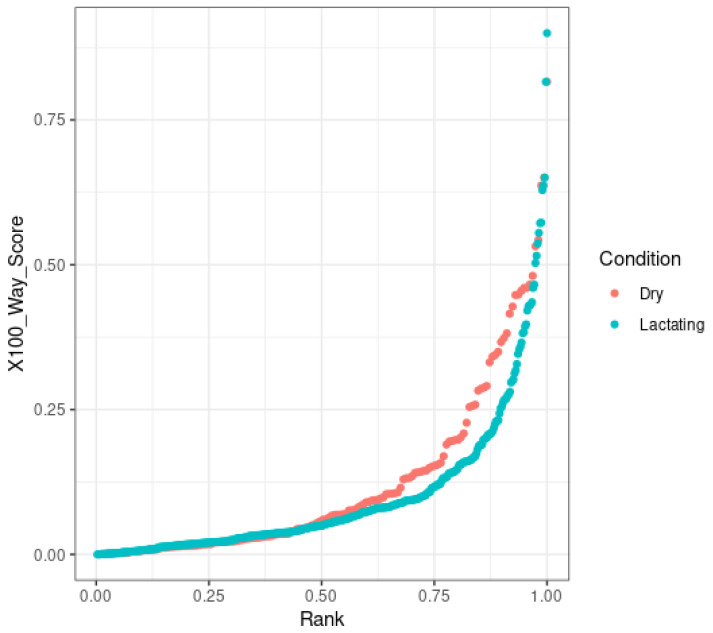
Scatter plot of lncRNA PhastCons scores. As is common, many candidate lncRNAs show very low sequence conservation with a small subset showing higher scores. Dry lncRNAs ranged from 0.001 to 0.816, with an average score of 0.12. Lactating lncRNA scores ranged from 0 to 0.9, with an average score of 0.1. These scores do not have a unit.

Of the reads successfully lifted over the human genome, the “all transcripts” group showed higher sequence conservation on average than the other two groups. Intergenic transcripts overall were more conserved than the lncRNA group but less than the “all transcripts” group. When plotted as a violin graph, it is readily apparent that most lncRNAs are localized closer to the 0 end of the scale, more so than the “all transcripts” group and the intergenic group. All transcripts scored between 0.0003 and 1, averaging 0.15. Intergenic dry transcripts scored between 0.00004 and 1 and averaged 0.16, with intergenic lactating transcripts scoring between 0 and 1 and averaging 0.15. Dry lncRNAs scored as low as 0.0001 and as high as 0.816, with an average of 0.12, and lactating lncRNAs scored between 0 and 0.9, averaging 0.1 (Figure 4). When plotted as rank versus score, it is obvious that most lncRNAs do not show high conservation, but a small amount are highly conserved (Figure 5). This falls in line with lncRNA trends [40]. Common lncRNAs scored between 0.00003 and 0.816, with an average score of 0.16, demonstrating that the common lncRNAs are slightly more conserved than all transcripts, intergenic transcripts, and all lncRNA transcripts.

### 3.5. Annotation of lncRNA of Interest Based on Homology

To attempt to functionally annotate lncRNA transcripts, sequence conservation was used to predict lncRNA roles. From the whole collection of lncRNAs, those with a conservation score of 0.5 or higher were kept for further analysis, as well as common lncRNAs with a conservation score of 0.25. Of the 214 dry lncRNAs, 5 transcripts were kept and 4 overlapped with annotated human genes; of the 517 lactating transcripts, 11 transcripts were kept and 9 overlapped with annotated human genes. The 106 common transcripts (53 from each sample) resulted in 28 kept transcripts, 12 from lactating transcripts, and 16 from dry transcripts, with 20 transcripts overlapping with an annotated human genetic feature. Several of the overlapped genetic features represented the same feature; of the 33 identified overlapped features, there were 18 unique features. Even the unique features tended to represent common functions: transcriptional regulation/factors, translational associations, signaling, and keratin filaments were all represented several times. Other roles featured in these conserved regions included inhibition of endothelial cell proliferation, association with synapse, inositol hydrolysis, and exocytosis regulation. Additionally, regions also overlapped with a known lncRNA and a documented pseudogene. The most common overlapped elements were as follows: DLG associated protein 2, keratin-associated protein 16-1, small cysteine and glycine repeat containing protein 1 and 2, and zinc finger homeobox protein 3. The use of gene ontology has associated DLG associated protein 2 with a role in signaling. Keratin-associated protein 16-1 is associated with keratin filament in the cytosol, as seen with gene ontology. The small cysteine and glycine repeat containing proteins (protein 1 and 2) are both matrix proteins that use extensive disulfide bond to cross-link with the cysteine residues of the hair keratins, leading to strong and resilient hair shafts. Although zinc finger homeobox protein 3 was seen more than other features, transcripts of interest also overlapped zinc finger protein 219 and zinc finger protein castor homolog 1; these features all have roles heavily associated with transcriptional regulation. Of the 9 transcripts not differentially expressed, 8 were successfully lifted over to represent human genome locations and analyzed for sequence conservation. Most of the transcripts showed low conservation; however, a small number showed conservation with a score above 0.25. Those with the slightly higher conservation showed overlap with adhesion G protein-coupled receptor L1, nuclear receptor coactivator 1, and disks large-associated protein 2. The functions of these proteins are a potential exocytosis regulator, a transcriptional coactivator, and a synapse associated protein, respectively.

### 3.6. lncRNA-Gene Coexpression Correlation and Ontology

Investigating lncRNA from another avenue, gene co-expression analysis was used to identify of genes expressed statistically significantly with a lncRNA. To avoid low expression based false correlations, any transcripts (lncRNAs or coding genes) with expression in less than 5 samples were removed and a correlation matrix was generated, yielding an R2 value for all pairwise relationships. For each lncRNA, those coding genes whose R2 value had a *p* value < 0.05 were isolated for gene ontology analysis. Although it is important to remember that correlation does not indicate causation, and showing statically significant correlation in their expression levels does not indicate that a lncRNA shares the same function as the correlated genes, the function of the gene cluster may be indicative of traits associated with the lncRNA. Like many lncRNA functional annotations, these act as reasonable hypotheses for lncRNA function. Of the 214 dry lncRNA, 126 were retained for correlation analysis. The expression levels of these lncRNAs were correlated with 29,809 coding genes, and R2 values ranged from −0.861 to 0.956, with the average correlation coefficient being 0.016, indicating there were not a substantial number of relationships that were more positively associated or more negatively associated. When investigating the clusters of statistically significantly correlated genes, many lncRNAs clustered with coding genes that showed some sort of function. In total, 15 of the 126 lncRNAs did not associate with any *Bos taurus* biological function, and the other 111 lncRNAs demonstrated statistically significant association with 85 unique functions. Several associations were related to regular bodily functions, such as cell differentiation, actin filament network formation, and immune responses; many associations represented regulation (in both the positive and negative direction) of cellular processes. The most highly represented association exists between lncRNAs and the regulation of telomere maintenance via telomerase, with this association representing 9 of the investigated clusters. Also associated with several lncRNAs were peptide antigen assembly with MHC class II protein complex (4 clusters), spindle assembly (3 clusters), regulation of natural killer cell mediated cytotoxicity (3 clusters), monoubiquitinated histone H2A deubiquitination (3 clusters), and the detection of chemical stimulus involved in sensory perception of smell (3 clusters). Of the 517 lactating lncRNAs, 233 were used for co-expression correlation analysis. These lncRNAs were correlated based on expression level with 24,374 coding genes. The reported R2 values fell in similar ranges to the dry samples, ranging from −0.870 to 0.950, with the average being 0.035, indicating the relationships skew slightly more in favor of positive correlations, but not in a notable manner. Whereas 11.9% of dry lncRNA-correlated clusters investigated showed no association, 22.3% of lactating lncRNA-correlated clusters did not return an acceptable association. Lactating lncRNA-associated clusters also represented many different connections, including cell maturation, protein insertions, immune response, and many instances of regulation. Clusters represented 122 unique associations, with the most highly represented cluster being the detection of chemical stimuli involved in sensory perception of smell, representing 12 clusters. Other repeatedly represented clusters include the regulation of telomere maintenance via telomerase (7 clusters), establishment of protein localization to mitochondrial membrane (5 clusters), and positive regulation of DNA biosynthetic processes (5 clusters).

### 3.7. lncRNA-Gene Coexpression Correlation Annotation

Using the smallest *p*-value to identify the most statistically significantly correlated gene for each lncRNA, several interesting associations and patterns emerged. Similarly to gene ontology analysis, correlation is not indicative of a guaranteed relationship, but clues to lncRNA function may reveal themselves. Of the 126 investigated lncRNAs in the dry tissue condition, 107 of the lncRNAs correlated with known named genes (the other 19 correlated with novel genes) and 103 of those known genes had functions/roles assigned to them. Correlated genes represent many roles, pathways, and functions. Gene functions that were represented by several genes were cell cycle regulation and/or cell proliferation, protein transport and binding, and various membrane interactions/features. A correlated gene of note is the MTA1 gene, which has been associated with mammary adenocarcinoma cells; although the role has not been fully investigated, the presence of this protein/gene may be useful in improving cattle health. A second notable gene associated with an identified lncRNA is the BTN1A1 gene, encoding a major protein that is associated with fat droplets in milk. The regulatory nature often seen in lncRNA makes this connection interesting; could this be used to continue the improvement of milk production? In lactating tissue, 233 lncRNAs were associated with genes. Of the 233 genes, 25 associated genes were novel transcripts, and 16 have been named and not fully investigated, and the remaining 192 have been assigned a role. Trends in associated gene function included protein binding, signaling, and mRNA and tRNA processing, as well as several cancer associations, although it should be noted that many roles, functions, and pathways were represented by these correlated genes. Noteworthy genes within lactating lncRNA correlations include: FOLR1, a folate receptor (folic acid, the receptor’s target, is very important in pregnancy); CAPN6, a protein highly expressed in the placenta; CSN3, a lactation-associated protein stabilizer; PTGFR, a protein implicated in uterus contractions; and OXTR, an oxytocin receptor. Given that pregnancy is the inciting event for the final mammary tissue development, the associations with pregnancy-related proteins and processes are worth further investigation. Harkening back to highly conserved lncRNAs and their potential function, KRT17 was an associated gene that yields the type I intermediate filament chain keratin 17, a protein found in nail beds, hair follicles, sebaceous glands, and other epidermal appendages. This correlation falls in line with the keratin-associated protein 16-1 and the small cysteine and glycine repeat containing proteins identified via sequence homology.

### 3.8. SNP Heritability Enrichment Analysis on Cattle Traits

To associate lncRNAs with traits of interest, the 214 dry tissue and 517 lactating tissue lncRNA transcripts identified were integrated with Holstein GWAS data to establish single nucleotide polymorphism (SNP) heritability enrichment for 4 traits: daughter pregnancy rate (to measure reproduction and fertility), livability (to measure health), milk production (to measure milk production value), and stature (to represent body conformation). Using MINQUE for partitioning heritability (MPH) (https://jiang18.github.io/mph/index (accessed on 1 June 2023)), SNP heritability enrichment was measured as a ratio of per-SNP heritability near the lncRNA of interest as compared to the entire genome. Enrichment analysis used 2,795,435 genome-wide SNPs, 4257 dry tissue SNPs, and 12,884 lactating tissue SNPs. As a result, significant enrichment of per-SNP heritability near lncRNAs was found in both dry and lactating tissues in reproduction- and production-associated traits. Enrichment was found to be significant in daughter pregnancy rate in dry tissue (8.92×; *p* = 0.015) and lactating tissue (4.96×; *p* = 0.019). Enrichment was also found to be significant in milk production, with dry tissue demonstrating an enrichment level of 7.39× (*p* = 0.01) and lactating tissue demonstrating an enrichment level of 4.73× (*p* = 0.003). Livability and stature were not statistically significantly enriched in either tissue condition (Table 2).

## 4. Discussion

This study aimed to collect publicly available RNA-Seq data from *Bos taurus* dry and lactating mammary tissue. The purpose was to identify lncRNAs present in each tissue type, identify those common to both conditions, and attempt to assign some sort of role or function to those lncRNA transcripts identified. Ideally, these findings will shed light on the role lncRNA plays in the development of the mammary gland as it develops to meet both the animal’s and our own needs. RNA-Seq data were collected from the NCBI SRA database before being aligned to a well-researched reference genome. Transcripts were filtered based on intergenic nature, coding potential, presence of protein domains, and sequence similarities to known genes, to narrow the transcripts down to a final list of 731 candidate lncRNA transcripts. When further analyzed, these transcripts showed shorter length, lower expression, fewer exons, and less sequence conservation than their whole genome counterparts; this is in line with previous lncRNA identification findings. These 731 transcripts represent 214 transcripts found in dry mammary tissue and 517 transcripts found in lactating tissue. Within these transcripts, 53 were identified to be common to both tissue conditions. The majority of these common transcripts were found to be statistically significantly differentially expressed, with 22 transcripts showing higher expression in dry tissue, 22 transcripts showing higher expression in lactating tissue, and 9 transcripts showing similar expression levels in both conditions. The identification of these lncRNAs created a framework of transcripts to further investigate and begin assessing potential functions and roles, as well as to surmise how these transcripts may fit into the development of the mammary tissue.

At the most basic level, there are lncRNA transcripts present in *Bos taurus* mammary tissue. The lncRNAs identified also varied in both presence and levels between dry and lactating samples, demonstrating that the genetic lncRNA profile is related to the changes that occur as a result of mammary gland development. To assign roles to the identified transcripts, sequence conservation was used to determine which transcripts were highly conserved and, therefore, more likely to act in a comparable way across species. Interestingly, many of the more conserved lncRNA elements tended to show high sequence conservation with documented transcriptional regulators. This supports previous research that lncRNA acts in regulatory fashions across the genome. The lncRNAs identified also varied in presence and levels between dry and lactating samples, showing that the genetic lncRNA profile is related to the changes that occur due to mammary gland development. Another common theme in elements showing high sequence conservation was proteins related to keratins; this is noteworthy because research has demonstrated that keratin intermediate filaments are crucial to physical resilience of epithelial tissues, especially in mammary glands [41]. Given the function of the mammary gland and the stress put on the tissue by nursing calves and the milking process, it is of benefit for a dairy cow to have strong keratin intermediate filaments, created by the strong epithelial cells in their mammary tissue.

Using gene co-expression and correlation to establish highly correlated lncRNA–gene relationships and clusters of genes that can be linked to a single lncRNA were also tools used to begin hypothesizing about transcript function. Although the correlation should not be mistaken for a definitive lncRNA function, a common school of thought indicates that genes with a positive correlation have effects that act in the same direction [42]. When investigating clusters of genes that correlated statistically significantly with lncRNAs, the functions and associations of these clusters were wide-reaching and cover many distinct types of roles. The associations seen between lncRNAs and general body function support the hypothesis that lncRNAs are crucial to the daily functioning of the animal. It is also unsurprising to see many associations between lncRNAs and the regulation of biological pathways, as lncRNAs are often heralded as regulatory elements. Most notably, however, is the association between gene clusters and sense of smell. lncRNA-correlated genes were in clusters associated with sense of smell in both dry and lactating tissues, but associated with more lncRNAs in the lactating tissue. Data collected and reviewed in human pregnancy revealed that many pregnant people report an increase in sensitivity to smell as their pregnancy progressed, so it is possible that this is occurring in cattle as well [43]. Given the inciting incident in lactation is pregnancy, the presence of these lncRNA-correlated gene clusters may contribute to changes in olfactory abilities in cattle as well. Investigating the single most significantly correlated gene for each lncRNA also yielded interesting results. Lactating lncRNAs showed several correlations with pregnancy-related genes as well. Genes relating to folic acid, oxytocin reception, uterus contractions, and lactation were all observed. Interestingly, keratin filament was again represented. When integrating GWAS results with lncRNAs to determine heritability of SNPs in relation to complex traits, enrichment was deemed to be significant in daughter pregnancy rate and milk production in both dry and lactating tissue. Interestingly, livability and stature were not statistically significantly enriched. These findings demonstrate the close relationship between mammary tissue lncRNAs and reproduction and production traits, as opposed to close relationships between all measured traits and these lncRNAs. The enrichment results for milk are of note as the function of the mammary gland is milk production; although gene changes have been well-investigated in the milk production pathway, lncRNAs are now being implicated in this pathway as well.

Although the use of public data allowed for a larger sample size than what may have been possible with live animal data collection, it does add variability into the analysis. This variability may allow for more rare variants to be collected, thus creating more in-depth findings; however, the increased variability could also lead to unintended noise within the data. The data being public also means that factors such as age, number of calving rounds, breeding history, and other health traits are outside of our control. Another potential source of irregularity may come from the development of the mammary gland and its relationship to the calving history of each specimen; does the genetic landscape of the animal look different when it is dry as a heifer versus between pregnancies? This is something that could be further investigated to generate a more thorough understanding of lncRNAs in this situation. The findings presented in this research project could be expanded in many different directions. The relationships between strong mammary epithelial tissue, keratin intermediate filaments, successful mammary development, and milking, and identified lncRNAs, could be useful to continue to develop healthy, well-producing animals. Both GWAS integrated results and co-expression correlation gene cluster ontology demonstrate that lncRNAs are associated with both complex traits and a large variety of biological paths and functions; therefore, lncRNA should not be ignored when investigating genetic components of biological processes.

## 5. Conclusions

In this study, dry and lactating mammary gland tissue were investigated for the presence of lncRNA. lncRNAs were found in both tissue conditions and demonstrated expected patterns for lncRNAs, supporting findings as true lncRNAs. lncRNA profiles differed between the two condition types with a small number of overlapping transcripts, contributing to the variable expression profiles and implicating lncRNAs in the morphological and biological changes that occur during lactation. Using various methods of lncRNA annotation, notable findings included strong hair keratin filaments, associations with the detection of chemical stimulus involved in sensory perception of smell, and enrichment of complex traits representing reproduction and production value. These findings imply that these lncRNAs are related to resilient mammary tissue, pregnancy-based changes, and complex traits of interest. The wide-reaching nature of these findings continues to add to the knowledge base and encourage the consideration of lncRNA in biologic research. 

## Figures and Tables

**Table 1:** *T*-test of expression levels between lncRNAs covering comparable regions of the genome, thus deemed to be the common lncRNAs. Average expression was calculated and reported in FPKM for each transcript. Two-sample *t*-tests and Welch’s *t*-tests were used to determine those transcripts expressed at levels deemed to be statistically significant based on a *p*-value of 0.05. 44 transcripts were deemed differentially expressed.

Dry		Lactating		*p*-Value
lncRNA	Expression	lncRNA	Expression	
chr1: 88,402,354–88,403,436	0.552	chr1: 88,402,258–88,403,346	0.436	0.2258
chr10: 28,036,993–28,037,708	7.006	chr10: 28,036,875–28,037,846	4.436	<$2.20\times 10^{-16}$
chr11: 102,644,124–102,648,416	0.592	chr11: 102,644,428–102,648,357	0.374	$4.48\times 10^{-11}$
chr11: 74,657,704–74,660,914	0.325	chr11: 74,658,354–74,660,959	0.281	0.06305
chr12: 32,163,152–32,163,433	0.953	chr12: 32,163,199–32,163,464	0.966	0.8596
chr12: 84,995,822–84,996,199	19.591	chr12: 84,995,788–84,996,023	16.090	0.03071
chr13: 29,201,242–29,205,645	0.305	chr13: 29,202,525–29,205,728	0.554	$1.80\times 10^{-10}$
chr13: 63,992,771–63,994,410	0.750	chr13: 63,992,252–63,994,789	0.310	$1.01\times 10^{-7}$
chr13: 76,610,578–76,612,313	0.617	chr13: 76,610,635–76,611,936	0.562	0.4465
chr14: 17,032–17,554	0.585	chr14: 16,830–18,164	0.733	0.001775
chr16: 42,802,614–42,812,241	33.971	chr16: 42,802,637–42,812,194	15.692	<$2.20\times 10^{-16}$
chr16: 48,586,547–48,586,896	0.458	chr16: 48,584,486–48,586,868	0.612	0.0005258
chr16: 71,804,283–71,805,220	1.440	chr16: 71,804,288–71,805,212	18.671	<$2.20\times 10^{-16}$
chr17: 69,703,586–69,705,950	2.227	chr17: 69,703,579–69,705,885	1.975	0.3815
chr18: 13,007,057–13,010,244	5.469	chr18: 13,007,179–13,009,811	28.722	$6.78\times 10^{-13}$
chr18: 1,829,126–1,830,665	0.196	chr18: 1,829,460–1,830,200	0.398	0.0001832
chr18: 38,105,619–38,111,753	6.826	chr18: 38,105,601–38,111,755	3.175	<$2.20\times 10^{-16}$
chr18: 58,480,168–58,480,492	0.326	chr18: 58,479,948–58,480,580	0.448	0.0004047
chr18: 7,018,326–7,020,326	0.521	chr18: 7,018,199–7,020,326	0.283	$2.41\times 10^{-08}$
chr19: 41,517,108–41,519,655	0.550	chr19: 41,518,998–41,519,227	0.288	$1.28\times 10^{-14}$
chr2: 111,874,235–111,875,148	4.087	chr2: 111,874,066–111,877,333	2.759	$1.96\times 10^{-5}$
chr2: 115,539,609–115,540,162	235.756	chr2: 115,539,562–115,540,164	138.488	$7.90\times 10^{-10}$
chr2: 125,783,114–125,794,043	24.285	chr2: 125,777,445–125,793,948	33.604	$3.36\times 10^{-9}$
chr21: 58,272,796–58,274,409	1.584	chr21: 58,272,808–58,274,430	0.854	<$2.20\times 10^{-16}$
chr21: 68,475,571–68,478,473	2.627	chr21: 68,475,747–68,477,894	2.432	0.6144
chr23: 11,415,564–11,420,435	19.060	chr23: 11,415,556–11,420,435	8.688	<$2.20\times 10^{-16}$
chr23: 17,153,445–17,154,227	8.728	chr23: 17,153,446–17,154,226	10.971	0.0002695
chr23: 17,771,969–17,776,464	2.292	chr23: 17,771,923–17,776,476	0.578	<$2.20\times 10^{-16}$
chr25: 1,035,522–1,036,687	10.186	chr25: 1,035,522–1,036,586	16.318	$5.80\times 10^{-15}$
chr25: 35,533,291–35,534,130	0.988	chr25: 35,533,317–35,534,096	19.412	<$2.20\times 10^{-16}$
chr25: 39,512,349–39,513,122	12.600	chr25: 39,512,418–39,513,043	5.562	<$2.20\times 10^{-16}$
chr25: 488,853–489,320	3.302	chr25: 488,852–489,379	2.741	0.02195
chr25: 786,947–787,788	1.176	chr25: 786,326–788,530	0.780	$3.55\times 10^{-8}$
chr26: 10,508,859–10,510,408	0.425	chr26: 10,508,507–10,510,415	0.526	$2.61\times 10^{-5}$
chr26: 21,485,237–21,487,166	0.418	chr26: 21,485,168–21,487,115	1.136	$2.49\times 10^{-11}$
chr26: 51,014,183–51,020,151	0.424	chr26: 51,016,793–51,020,761	0.234	$1.81\times 10^{-14}$
chr27: 1,092,242–1,093,287	0.894	chr27: 1,092,247–1,093,275	1.036	0.08457
chr29: 46,399,007–46,401,176	0.316	chr29: 46,399,026–46,401,485	0.302	0.745
chr29: 46,623,288–46,624,946	1.844	chr29: 46,623,270–46,625,478	3.484	$1.86\times 10^{-9}$
chr29: 47,279,192–47,281,419	0.440	chr29: 47,278,057–47,281,321	1.610	<$2.20\times 10^{-16}$
chr29: 47,437,521–47,440,208	0.603	chr29: 47,437,552–47,440,146	1.012	$3.96\times 10^{-12}$
chr4: 52,093,644–52,094,267	4.437	chr4: 52,093,643–52,094,277	0.438	$1.16\times 10^{-14}$
chr5: 118,095,776–118,100,684	0.434	chr5: 118,095,874–118,100,834	0.303	$9.99\times 10^{-5}$
chr6: 3,400,389–3,401,869	0.596	chr6: 3,400,394–3,401,718	5.301	<$2.20\times 10^{-16}$
chr6: 62,052,709–62,053,912	0.368	chr6: 62,052,696–62,054,059	0.511	0.002736
chr7: 10,013,140–10,014,516	1.509	chr7: 10,013,140–10,014,265	0.391	$2.82\times 10^{-8}$
chr7: 11,559,071–11,560,742	0.058	chr7: 11,559,861–11,561,374	0.100	0.2047
chr7: 17,181,130–17,183,216	0.703	chr7: 17,181,196–17,183,208	11.387	<$2.20\times 10^{-16}$
chr7: 60,229,378–60,232,948	9.270	chr7: 60,229,471–60,232,796	4.910	$7.91\times 10^{-15}$
chr9: 98,186,197–98,187,338	1.152	chr9: 98,186,218–98,187,339	3.450	<$2.20\times 10^{-16}$
ChrUn.004.1159: 35,363–37,180	0.171	ChrUn.004.1159: 35,361–38,038	0.344	$3.20\times 10^{-5}$
ChrUn.004.301: 120,666–127,066	0.410	ChrUn.004.301: 124,684–128,068	0.170	$6.17\times 10^{-15}$
ChrUn.004.3283: 1785–2847	0.330	ChrUn.004.3283: 1822–2792	2.147	$8.56\times 10^{-13}$

**Table 2:** Enrichment of mammary lncRNAs in both dry and lactating tissue, based on integrated cattle GWAS data using the MPH software. Daughter pregnancy rate (DPR) represents reproduction value, livability represents health, milk represents production value, and stature represents body conformation. Data reported include estimate of per-SNP heritability enrichment, standard error of estimate of per-SNP heritability enrichment estimate, and *p*-value for per-SNP heritability enrichment estimate.

	DPR			Livability			Milk			Stature		
	**Enr**	**SE**	***p***	**Enr**	**SE**	***p***	**Enr**	**SE**	***p***	**Enr**	**SE**	***p***
**Dry**	8.92	3.65	0.02	6.10	5.12	0.16	7.39	2.50	0.01	2.93	2.27	0.20
**Lact**	4.96	1.91	0.02	−0.39	2.39	0.72	4.73	1.36	$3.00\times 10^{-3}$	6.10	1.53	0.10

## Data Availability

All data were collected from the SRA database using search terms such as “*Bos taurus*”, “mammary tissue”, and “lactating”. SRA accession numbers available in Supplemental Table S1.

## Supplementary Materials

The following supporting information can be downloaded at: https://www.mdpi.com/article/10.3390/genes14091789/s1, Table S1: SRR IDs for all samples used in the analysis.
