# Supplementary material for: Investigation of lncRNA in Bos taurus Mammary Tissue during Dry and Lactation Periods
Source: Genes (Basel). 2023 Sep 12;14(9):1789. doi: 10.3390/genes14091789 (PMC10531232; doi:10.3390/genes14091789)
Supplement: Supplementary file 1 [file genes-14-01789-s001.zip › genes-2578085-supplementary.pdf]

Supplementary Table S1

Alexis Marceau

08/09/2023

Supplementary Table S1: SRR IDs for all samples used in this analysis.

Table S1:

**Dry**	**ID**	**Lactating**	**ID**
Dry	SRR6448594	Lactating	SRR13247961
Dry	SRR6448595	Lactating	SRR13247962
Dry	SRR6448596	Lactating	SRR13247963
Dry	SRR6448597	Lactating	SRR13247964
Dry	SRR6448598	Lactating	SRR13247965
Dry	SRR6448599	Lactating	SRR13247966
Dry	SRR6448600	Lactating	SRR13247967

Continued on next page

Table S1: (Continued)

**Dry**	**ID**	**Lactating**	**ID**
Dry	SRR6448601	Lactating	SRR13247968
Dry	SRR6448602	Lactating	SRR13247969
Dry	SRR6448603	Lactating	SRR13247970
Dry	SRR6448604	Lactating	SRR13247971
Dry	SRR6448605	Lactating	SRR13247972
Dry	SRR6448606	Lactating	SRR13247973
Dry	SRR6448607	Lactating	SRR13247974
Dry	SRR6448608	Lactating	SRR13247975
Dry	SRR6448609	Lactating	SRR13247976
Dry	SRR6448610	Lactating	SRR13247977
Dry	SRR6448611	Lactating	SRR13247978
Dry	SRR6448612	Lactating	SRR13247979
Dry	SRR6448613	Lactating	SRR13247980
Dry	SRR6448614	Lactating	SRR13247981
Dry	SRR6448615	Lactating	SRR13247982
Dry	SRR6448616	Lactating	SRR13247983
Dry	SRR6448617	Lactating	SRR13247984

Continued on next page

Table S1: (Continued)

Dry	ID	Lactating	ID
Dry	SRR6448618	Lactating	SRR13247985
Dry	SRR6448619	Lactating	SRR13247986
Dry	SRR6448620	Lactating	SRR13247987
Dry	SRR6448621	Lactating	SRR13247988
Dry	SRR6448622	Lactating	SRR13247989
Dry	SRR6448623	Lactating	SRR13247990
Dry	SRR6448624	Lactating	SRR13247991
Dry	SRR6448625	Lactating	SRR13247992
Dry	SRR6448626	Lactating	SRR13247993
Dry	SRR6448627	Lactating	SRR13247994
Dry	SRR6448628	Lactating	SRR13247995
Dry	SRR6448629	Lactating	SRR13247996
Dry	SRR6448630	Lactating	SRR13247997
Dry	SRR6448631	Lactating	SRR13247998
Dry	SRR6448632	Lactating	SRR13247999
Dry	SRR6448633	Lactating	SRR13248000
Dry	SRR6448634	Lactating	SRR13248001

Continued on next page

Table S1: (Continued)

Dry	ID	Lactating	ID
Dry	SRR6448635	Lactating	SRR13248002
Dry	SRR6448636	Lactating	SRR13248003
Dry	SRR6448637		
Dry	SRR6448638		
Dry	SRR6448639		
Dry	SRR6448640		
Dry	SRR6448641		
Dry	SRR6448642		
Dry	SRR6448643		
Dry	SRR6448644		
Dry	SRR6448645		
Dry	SRR6448646		
Dry	SRR6448647		
Dry	SRR6448648		
Dry	SRR6448649		
Dry	SRR6448650		
Dry	SRR6448651		

Continued on next page

Table S1: (Continued)

Dry	ID	Lactating	ID
Dry	SRR6448652		
Dry	SRR6448653		
